# Splicing factor PRP-19 regulates mitochondrial stress response

**DOI:** 10.1093/lifemeta/loac009

**Published:** 2022-06-24

**Authors:** Peixue Xia, Liankui Zhou, Jialiang Guan, Wanqiu Ding, Ying Liu

**Affiliations:** State Key Laboratory of Membrane Biology, Institute of Molecular Medicine, College of Future Technology, Peking University, Beijing, China; Peking-Tsinghua Center for Life Sciences, Peking University, Beijing 100871, China; State Key Laboratory of Membrane Biology, Institute of Molecular Medicine, College of Future Technology, Peking University, Beijing, China; Peking-Tsinghua Center for Life Sciences, Peking University, Beijing 100871, China; PKU-Tsinghua-NIBS Graduate Program, Academy for Advanced Interdisciplinary Studies, Peking University, Beijing 100871, China; State Key Laboratory of Membrane Biology, Institute of Molecular Medicine, College of Future Technology, Peking University, Beijing, China; State Key Laboratory of Membrane Biology, Institute of Molecular Medicine, College of Future Technology, Peking University, Beijing, China; Peking-Tsinghua Center for Life Sciences, Peking University, Beijing 100871, China; Beijing Advanced Innovation Center for Genomics, Beijing, China

**Keywords:** mitochondria, unfolded protein response, stress response, splicing, lifespan

## Abstract

Animals respond to mitochondrial perturbation by activating the mitochondrial unfolded protein response (UPR^mt^) to induce the transcription of mitochondrial stress response genes. In *Caenorhabditis elegans*, activation of UPR^mt^ allows the animals to maintain organismal homeostasis, activate the innate immune response, and promote lifespan extension. Here, we show that splicing factors such as Precursor RNA processing 19 (PRP-19) are required for the induction of UPR^mt^ in *C. elegans*. PRP-19 also modulates mitochondrial perturbation-induced innate immune response and lifespan extension. Knockdown of PRP-19 in mammalian cells suppresses UPR^mt^ activation and disrupts the mitochondrial network. These findings reveal an evolutionarily conserved mechanism that maintains mitochondrial homeostasis and controls innate immunity and lifespan through splicing factors.

## Introduction

Living organisms actively face challenges from the ever-changing environment. The ability to sense and respond to environmental changes is critical for organismal survival. As essential cellular organelles, mitochondria are constantly challenged by intrinsic stimuli such as reactive oxygen species (ROS) and extrinsic pathogens or xenobiotics [1]. Failure to respond to mitochondrial stresses can result in multiple diseases including neurodegenerative disorders [2, 3].

In *Caenorhabditis elegans*, mitochondrial perturbation activates a surveillance program named the mitochondrial unfolded protein response (UPR^mt^), which initiates mitochondrion-to-nucleus communication to elevate the transcription of genes encoding mitochondrial chaperones and proteases, thereby buffering the mitochondrial folding environment [4, 5]. UPR^mt^ also increases animal fitness by eliciting metabolic reprogramming [6], increasing innate immunity, and promoting longevity [1, 7–11]. In *C. elegans*, the transcriptional regulation of UPR^mt^ genes is mainly regulated through two axes driven by ATFS-1 or DVE-1 [10]. ATFS-1 is a transcription factor that contains an N-terminal mitochondrial targeting sequence and a C-terminal nuclear localization sequence. Under normal conditions, ATFS-1 is imported into mitochondria and degraded by the mitochondrial protease LONP-1. Upon mitochondrial perturbation, the impaired mitochondrial import results in the nuclear accumulation of ATFS-1 and elevated transcription of UPR^mt^ genes [12]. DVE-1 is a homeobox protein homologous to human SATB1/SATB2, which functions as a genome organizer. During mitochondrial stress, DVE-1 accumulates in the nucleus and coordinates with histone deacetylase HDA-1 to promote the transcription of UPR^mt^ genes [9, 11, 13].

Spliceosome is a dynamic RNA–protein complex composed of five core small nuclear ribonucleoprotein particles (snRNPs) and around 100 cofactors. It removes the non-coding sequences (introns) and ligates the coding sequences (exons) in precursor messenger RNA (pre-mRNA) [14–16]. The Precursor RNA processing 19 (Prp19) complex, also known as NineTeen Complex (NTC), plays an important role in the catalytic activation of spliceosome [17]. Spliceosomes are highly conserved across species. Interestingly, recent studies have shown that the splicing machinery can regulate gene expression through a splicing-independent function by affecting transcription and chromatin remodeling [18–20]. For instance, splicing factors can interact with RNA Polymerase II C-terminal domain to promote transcription elongation [21, 22].

In this study, we explore the effect of splicing factors on UPR^mt^ in a physiologically relevant setting. We show that upon mitochondrial perturbation, splicing factors such as PRP-19 are required for the induction of UPR^mt^ in *C. elegans*. PRP-19 also plays an essential role in promoting innate immunity and lifespan extension under mitochondrial stress conditions. Moreover, PRPF19, the mammalian ortholog of PRP-19, regulates mitochondrial homeostasis in mammals. In summary, our results reveal an essential function of splicing factors in UPR^mt^ signaling.

## Results

### Splicing factor PRP-19 is required for the activation of UPR^mt^

**Figure 1:**
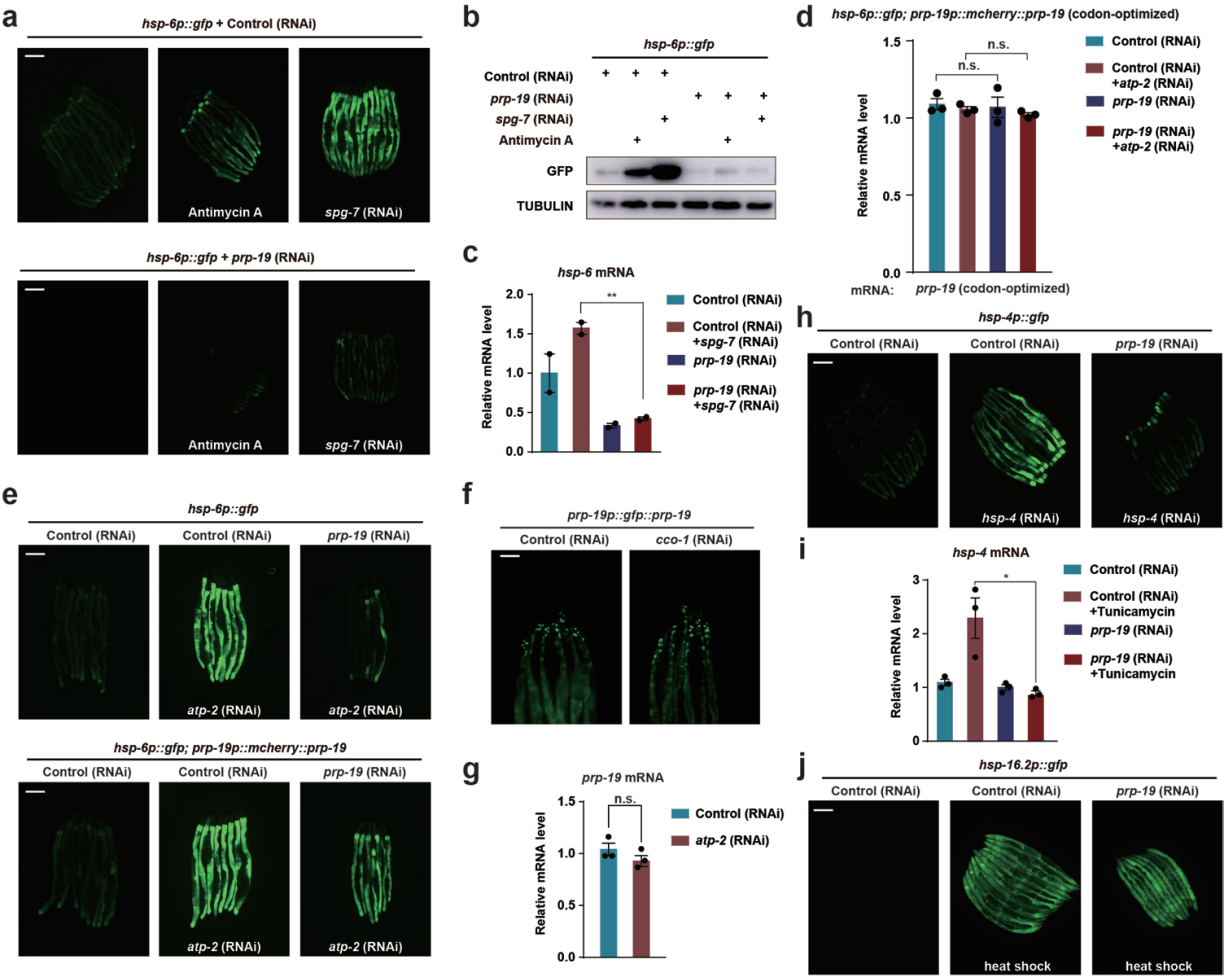
*prp-19* is required for UPR^mt^ activation. (a) Fluorescence images of *hsp-6p::gfp* worms at day 1 adulthood. Top: *hsp-6p::gfp* worms were fed on control RNAi and then treated or untreated with Antimycin A or *spg-7* RNAi. Bottom: *hsp-6p::gfp* worms were fed on *prp-19* RNAi and then treated with or without Antimycin A or *spg-7* RNAi. Scale bar, 200 µm. (b) Immunoblotting of GFP protein level in *hsp-6p::gfp* worms. (c) Quantitative RT-PCR of endogenous *hsp-6* mRNA levels in *glp-4(bn2)* fed on control or *prp-19* RNAi and treated with or without *spg-7* RNAi, **P < .005. (d) Quantitative RT-PCR of codon-optimized transgenic *prp-19* mRNA levels in *hsp-6p::gfp*; *prp-19p::mcherry::prp-19* raised on control or *prp-19* RNAi and treated with control or *atp-2* RNAi. n.s., no significance. (e) Fluorescence images of *hsp-6p::gfp* (top) or *hsp-6p::gfp*; *prp-19p::mcherry::prp-19* (bottom) animals grown on control or *prp-19* RNAi. Worms were then untreated or treated with *atp-2* RNAi. Overexpressed *prp-19* was codon optimized. Scale bar, 200 µm. (f) Fluorescence images of *prp-19p::gfp::prp-19* animals grown on control or *cco-1* RNAi. Scale bar, 100 µm. (g) Quantitative RT-PCR of endogenous *prp-19* mRNA levels in N2 worms raised on control or *atp-2* RNAi. (h) Fluorescence images of *hsp-4p::gfp* at day 1 adulthood. *hsp-4p::gfp* worms grown on control or *prp-19* RNAi. Worms were then treated with or without *hsp-4* RNAi. (i) Quantitative RT-PCR of endogenous *hsp-4* mRNA levels in control or tunicamycin-treated animals fed with control or *prp-19* RNAi. *P < .05. (j) Fluorescence images of *hsp-16.2p::gfp* at day 1 adulthood. *hsp-16.2p::gfp* worms grown on control or *prp-19* RNAi, followed by heat shock treatment. Scale bar, 200 µm.

We previously completed a genome-wide RNAi screen to identify genes required for the induction of UPR^mt^ in *C. elegans* [1]. Precursor RNA processing 19 (*prp-19*), a gene with an essential role in splicing in *C. elegans*, is one of the hits from our screen. Knockdown of *prp-19* by RNAi suppressed the activation of UPR^mt^ induced by Antimycin A, a mitochondrial electron transport chain (ETC) complex III inhibitor, or RNAi of the nucleus-encoded mitochondrial metalloprotease gene *spg-7* (Fig. 1a and b). In worms with mitochondrial perturbation induced by *spg-7* RNAi, RNAi of *prp-19* also impaired the expression and induction of the endogenous mitochondrial stress response gene *hsp-6* (Fig. 1c). To exclude the possibility that *prp-19* RNAi may generate off-target effects, we overexpressed a codon-optimized PRP-19, the expression of which could not be suppressed by *prp-19* RNAi (Fig. 1d). Overexpression of PRP-19 in *prp-19* RNAi worms restored the induction of UPR^mt^ (Fig. 1e). PRP-19 is mainly expressed in the nuclei of intestinal cells (Fig. 1f). The subcellular localization and the expression level of PRP-19 were not affected by mitochondrial perturbation (Fig. 1f and g). Notably, *prp-19* RNAi also affected the induction of the endoplasmic reticulum (ER) stress reporter *hsp-4p::gfp* when the worms were challenged with *hsp-4* RNAi or tunicamycin (Fig. 1h and i). Conversely, the heat shock response and the induction of *hsp-16.2p::gfp* reporter were not affected by knockdown of *prp-19* (Fig. 1j).

**Figure 2:**
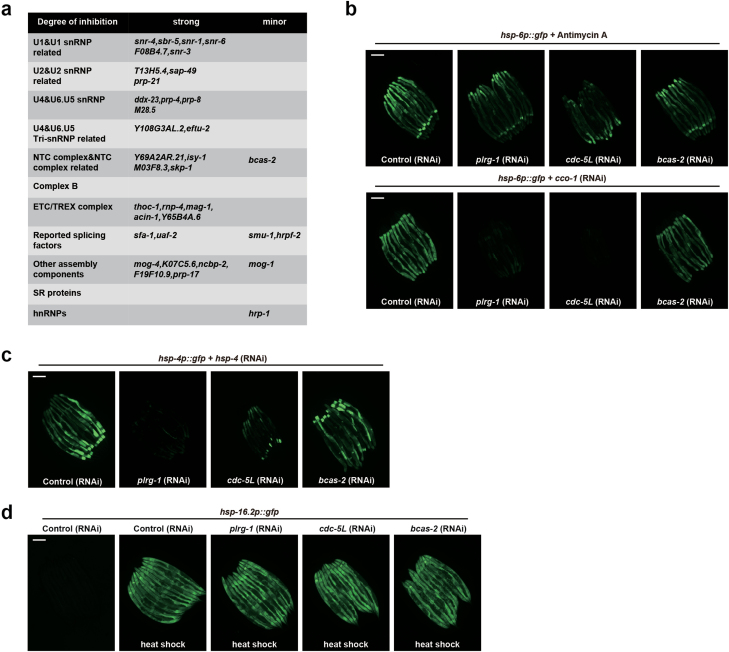
Splicing factors are required for UPR^mt^ activation. (a) List of splicing factors tested that affect the activation of UPR^mt^. (b) *hsp-6p::gfp* animals raised on the indicated RNAi in the presence or absence of Antimycin A or *cco-1* RNAi. Scale bar, 200 µm. (c) *hsp-4p::gfp* animals raised on the indicated RNAi in the presence of *hsp-4* RNAi. Scale bar, 200 µm. (d) Fluorescence images *of hsp-16.2p::gfp* animals raised on the indicated RNAi followed by heat shock at 37°C for 1 h. Scale bar, 200 µm.

PRP-19 is a core component of the large Prp19C/NTC complex mentioned above. This protein complex consists of 8 core proteins and more than 30 other proteins in mammals [23]. The best characterized function of Prp19C/NTC is its role in the catalytic activation of the spliceosome [17, 24]. Prp19C/NTC has also been shown to play a role in transcription elongation and the maintenance of genome stability [25–27]. To see if other splicing factors similarly modulate UPR^mt^, we individually knocked down a broad spectrum of splicing factors in different spliceosome subunits, including many splicing factors functioning outside of the Prp19C/NTC complex (Fig. 2a). RNAi of most of the splicing factors strongly suppressed the induction of UPR^mt^ and UPR^ER^, but not the heat shock response (Fig. 2b–d). These results suggest that PRP-19 regulates UPR^mt^ activation through its function in splicing.

Alternative splicing (AS) is a post-transcriptional process in eukaryotes that produces different isoforms of mRNAs and increases the diversity of gene expression. AS events are divided into five main patterns: use of alternative 3ʹ (acceptor) splice sites (A3SS), use of alternative 5ʹ (donor) splice sites (A5SS), mutually exclusive exon (MXE) usage, retention of introns (RI), and skipping of exons (SE) [28]. To test if any AS events are required for the production of factors that play a role in UPR^mt^ regulation, we performed RNA-seq experiments to detect global transcriptome alteration. Mitochondrial perturbation induced by *atp-2* RNAi only caused 8 alternatively spliced transcripts in total, whereas knockdown of *prp-19* alone or knockdown of *prp-19* together with *atp-2* resulted in 117 or 102 AS events, respectively (Supplementary Fig. S1a and b and Supplementary Table S1). We validated some of the AS events and confirmed the RNA-seq results (Supplementary Fig. S1c–f). However, UPR^mt^ genes were not alternatively spliced (Supplementary Table S1). We also found no evidence that alternatively spliced genes play a role in UPR^mt^ activation.

### PRP-19 functions downstream of ATFS-1 to modulate mitochondrial stress response

To explore the molecular mechanism by which PRP-19 modulates UPR^mt^, we tested if knockdown of *prp-19* affected the functions of known components in the UPR^mt^ pathway. In *C. elegans*, the transcriptional induction of UPR^mt^ is mainly governed by two transcription factors, ATFS-1 and DVE-1 [10, 12, 13]. In addition, a histone deacetylase HDA-1 functions in concert with DVE-1 to activate the transcription of UPR^mt^ genes [11]. Unlike *hda-1* RNAi, which reduces the DVE-1 protein level [11], knockdown of *prp-19* actually promoted the nuclear accumulation of DVE-1 and elevated the DVE-1 protein level (Supplementary Fig. S2a). Consistent with this, *prp-19* RNAi also promoted the nuclear accumulation of HDA-1 and elevated the HDA-1 protein level (Supplementary Fig. S2b and c). Moreover, knockdown of *prp-19* promoted the interaction between DVE-1 and HDA-1 (Supplementary Fig. S2d). Collectively, these results indicate that PRP-19 may not regulate UPR^mt^ gene expression through DVE-1 and HDA-1.

**Figure 3:**
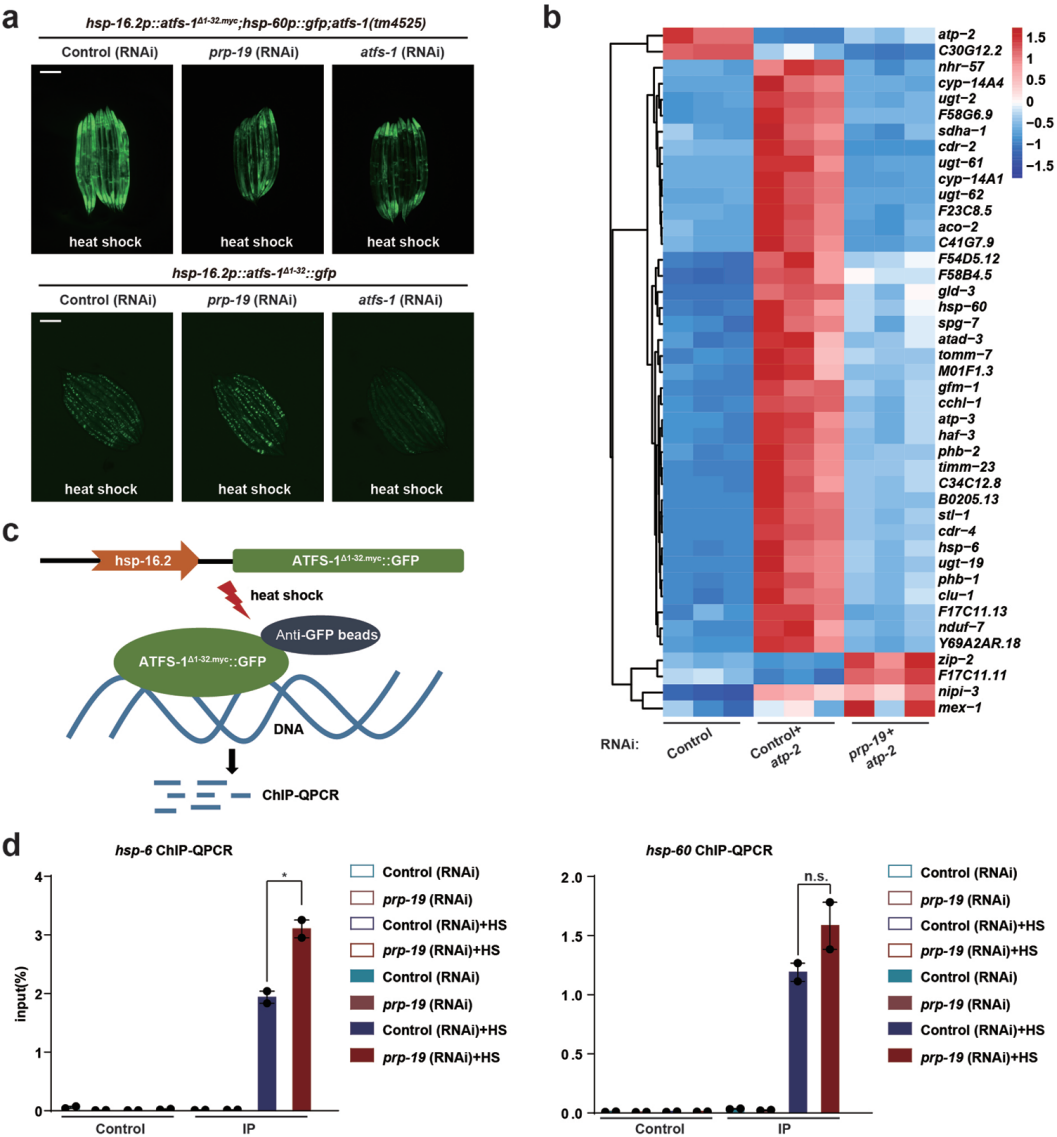
PRP-19 functions downstream of ATFS-1. (a) Fluorescence images of *hsp-16.2p::atfs-1*^*Δ1-32.myc*^; *hsp-60p::gfp*; *atfs-1(tm4525)* (top) or *hsp-16.2p::atfs-1*^*Δ1-32*^::*gfp* (bottom) animals raised on control, *prp-19* or *atfs-1* RNAi, and followed by heat shock treatment. Scale bar, 200 µm. (b) Heat map of the expression levels of the *prp-19*-dependent genes whose promoters are bound by ATFS-1 during mitochondrial stress. log_2_(fold change) > 1, *P* < .05. (c) Diagram of the ATFS-1 ChIP assay. (d) Anti-GFP ChIP-QPCR of *hsp-6* or *hsp-60* promoters. *hsp-16.2p::atfs-1*^*Δ1-32.myc*^*::gfp* worms were raised on control or *prp-19* RNAi, followed by heat shock treatment to induce the expression of ATFS-1^Δ1-32.myc^::GFP. *n* = 2, *P < 0.05. n.s., no significance.

We then tested if PRP-19 modulates UPR^mt^ gene expression via ATFS-1. The transcription factor ATFS-1 harbors an amino-terminal mitochondrial targeting sequence and a carboxy-terminal nuclear localization sequence. Upon mitochondrial perturbation, the import efficiency of mitochondria is decreased, leading to the nuclear accumulation of ATFS-1 [12]. Deletion of the N-terminal amino acids 1–32 of ATFS-1 disrupts its mitochondrial targeting signal, resulting in constitutive nuclear accumulation of ATFS-1 and activation of UPR^mt^ gene expression [12]. We employed a transgenic strain that allows the expression of ATFS-1^Δ1-32.myc^ upon heat shock and noticed that expression of the UPR^mt^ gene *hsp-60* also requires *prp-19* (Fig. 3a). Knockdown of *prp-19* did not affect the expression and nuclear accumulation of ATFS-1^Δ1-32^ after heat shock (Fig. 3a), suggesting that PRP-19 acts downstream of ATFS-1 once it enters the nucleus.

After entering the nucleus, ATFS-1 binds to the promoter of UPR^mt^ genes and activates their transcription. A group of ATFS-1-dependent UPR^mt^ genes, including mitochondrial chaperone genes *hsp-6* and *hsp-60*, mitochondrial import complex genes *tomm-7* and *timm-23*, and innate immune response genes *cyp-14A4* and *ugt-61*, also require the presence of PRP-19 for their induction during mitochondrial stress (Fig. 3b). We therefore sought to test if lack of PRP-19 affects the binding of ATFS-1 to the promoters of UPR^mt^ genes. Again, we employed a transgenic strain which expresses ATFS-1^Δ1-32.myc^::GFP upon heat shock induction. Chromatin immunoprecipitation (ChIP) coupled with quantitative PCR (qPCR) was performed using anti-GFP antibody to pull-down ATFS-1^Δ1-32.myc^::GFP (Fig. 3c). A significant amount of ATFS-1^Δ1-32.myc^ was associated with the promoters of *hsp-6* and *hsp-60* after heat shock induction. However, knockdown of *prp-19* did not impair, but rather promoted the binding of ATFS-1 to the promoters of *hsp-6* (Fig. 3d). Therefore, PRP-19 and spliceosome seem to function in a step after ATFS-1 associates with the promoter of mitochondrial stress response genes to modulate UPR^mt^ activation.

Interestingly, a recent study has shown that spliceosomal repression directly affects gene transcription in mouse embryonic stem cells (ESCs), resulting in decreased expression of pluripotent genes, but not affecting the expression of totipotent genes [29]. This study proposed that the greater length and increased number of introns in pluripotent genes relative to totipotent genes may explain their transcriptional regulation by spliceosomal repression. However, we analyzed the number and length of introns in the UPR^mt^ genes and noticed no difference compared with other genes not regulated by the mitochondrial stress response (data not shown). Therefore, at this stage, the detailed mechanism of how the transcription of UPR^mt^ genes is specifically regulated by the splicing factors remains elusive.

### PRP-19 is essential for UPR^mt^-mediated innate immunity and lifespan extension

**Figure 4:**
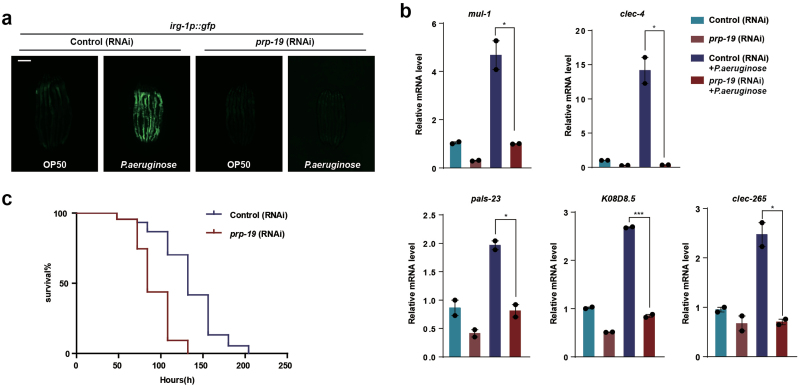
PRP-19 regulates UPR^mt^-mediated innate immune response. (a) Fluorescence images of *irg-1p::gfp* worms raised on control or *prp-19* RNAi in the presence of OP50 or *P. aeruginosa*. (b) qPCR measurement of endogenous *mul-1*, *clec-4*, *pals-23*, *K08D8.5*, and *clec-265* mRNA levels in worms fed on control or *prp-19* RNAi and treated with OP50 or *P. aeruginosa*. *P < .05, ***P < .0005. (c) *Pseudomonas aeruginosa* slow-killing assay of *glp-4(bn2)* worms raised on control or *prp-19* RNAi. *n* = 3 biological replicates. *n* >100 worms for each condition.

Mitochondrial function is actively challenged by wild microbes in the natural habitats of *C. elegans* [1]. As a defense mechanism, UPR^mt^ also initiates the innate immune response [1, 7] and promotes lifespan extension [8, 10, 11]. Therefore, we further tested the function of PRP-19 in innate immunity and lifespan regulation. *irg-1p::gfp* has been used as a reporter strain for the induction of innate immune response [1, 30]. We challenged the *irg-1p::gfp* transgenic worms with a *Pseudomonas aeruginosa* strain isolated from natural habitats of *C. elegans*. This pathogen has been shown to disrupt mitochondrial function and activate UPR^mt^ [1]. Knockdown of *prp-19* suppressed the induction of *irg-1p::gfp* upon pathogen infection (Fig. 4a). Deficiency of *prp-19* also suppressed the endogenous induction of several other immune response genes and reduced the survival rate of *C. elegans* when they were exposed to *Pseudomonas aeruginosa* (Fig. 4b and c). Taken together, these results indicate that PRP-19 plays a critical role in mediating the innate immune response.

**Figure 5:**
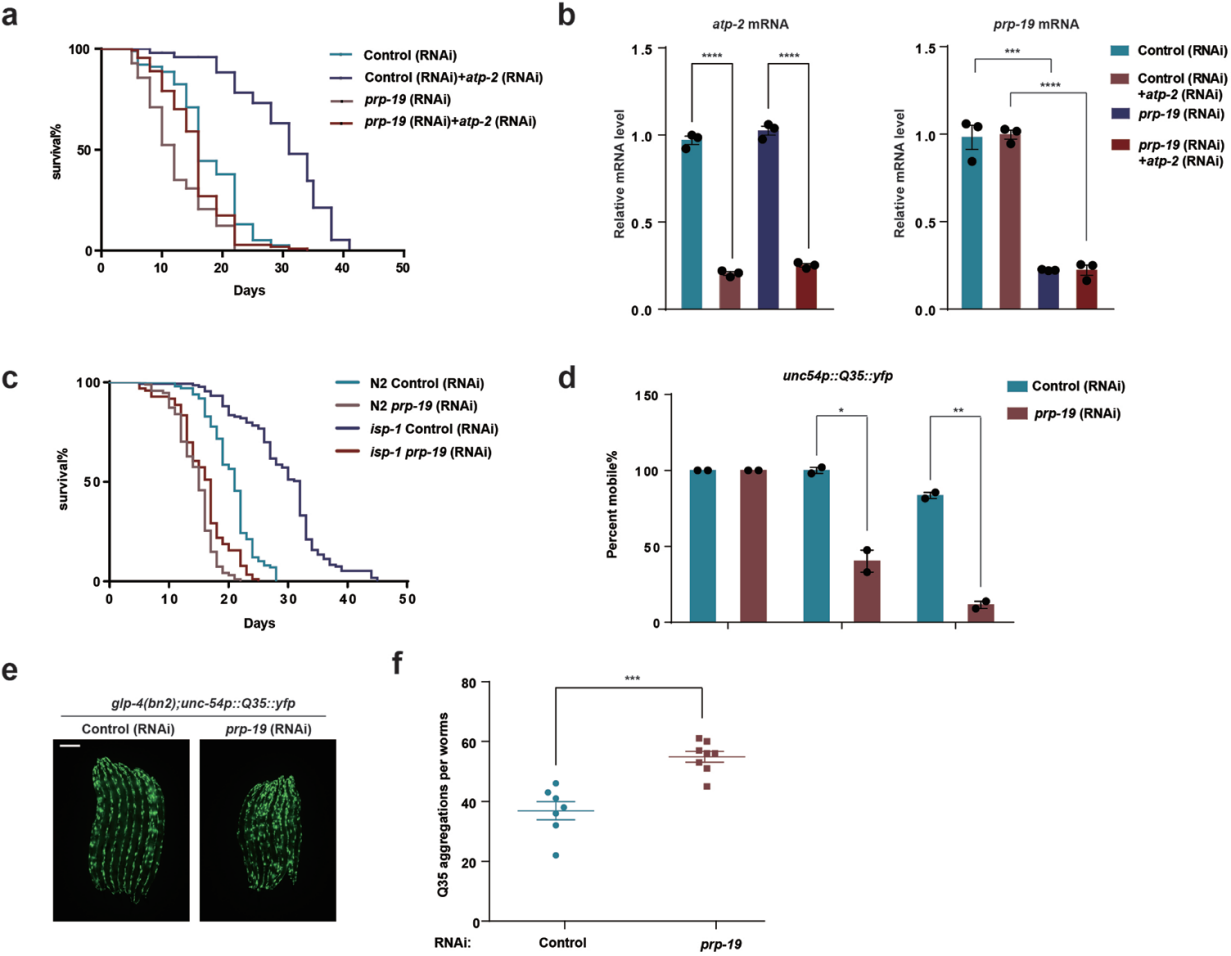
PRP-19 regulates UPR^mt^-mediated lifespan extension. (a) Lifespan of animals raised on control or *prp-19* RNAi in the presence or absence of *atp-2* RNAi. *n* = 2 biological replicates. *n* >50 worms for each condition. (b) qPCR measurement of endogenous *atp-2* and *prp-19* mRNA levels in worms raised on control or *prp-19* RNAi and fed with control or *atp-2* RNAi. ***P < .0005, ****P < .0001. (c) Lifespan of wild-type N2 worms or *isp-1* mutant worms raised on control or *prp-19* RNAi. *n* = 2 biological replicates. *n* > 100 worms for each condition. (d) Mobility analysis of *unc-54p::Q35::yfp* worms grown on control or *prp-19* RNAi on day 1, day 7 or day 11 of adulthood. *n* = 2 biological replicates, *n* > 80 worms per condition. *P < .05, **P < .005. (e) Fluorescence images of Q35::YFP aggregates of *glp-4(bn2)*, *unc-54p::Q35::yfp* worms raised on control or *prp-19* RNAi on day 5 of adulthood. Scale bar, 200 µm. (f) The number of Q35::YFP aggregates per worm under control or *prp-19* RNAi conditions. *n* = 7, 8. ***P < .0005.

The physiological function of PRP-19 was assessed by examining lifespan regulation in the presence or absence of mitochondrial stress. Under normal conditions, *prp-19* RNAi had a minor shortening effect on worm lifespan (Fig. 5a). However, knockdown of *prp-19* greatly suppressed the lifespan extension in *atp-2* RNAi-treated worms (Fig. 5a). To test the knockdown efficiency of double RNAi, we performed qPCR experiments and showed that the reduction in *atp-2* expression achieved with a mix of *atp-2* RNAi plus *prp-19* RNAi is similar to that with *atp-2* RNAi plus the control RNAi (Fig. 5b). In addition, knockdown of *prp-19* suppressed the lifespan extension of worms carrying a mutation in the mitochondrial gene *isp-1* (Fig. 5c). Age-related decline of protein homeostasis results in the toxic accumulation of protein aggregates, including aggregates of proteins containing polyglutamine (polyQ) repeats [2, 11]. Consistent with the role of PRP-19 in lifespan regulation, knockdown of *prp-19* impaired the mobility of worms expressing polyQ repeats (Fig. 5d) and significantly increased the number of polyQ aggregates in *C. elegans* (Fig. 5e and f). Collectively, these results suggest that PRP-19 is essential for the UPR^mt^-mediated beneficial impact to alleviate age-related pathology.

### PRP-19 modulates mitochondrial stress response in mammals

**Figure 6:**
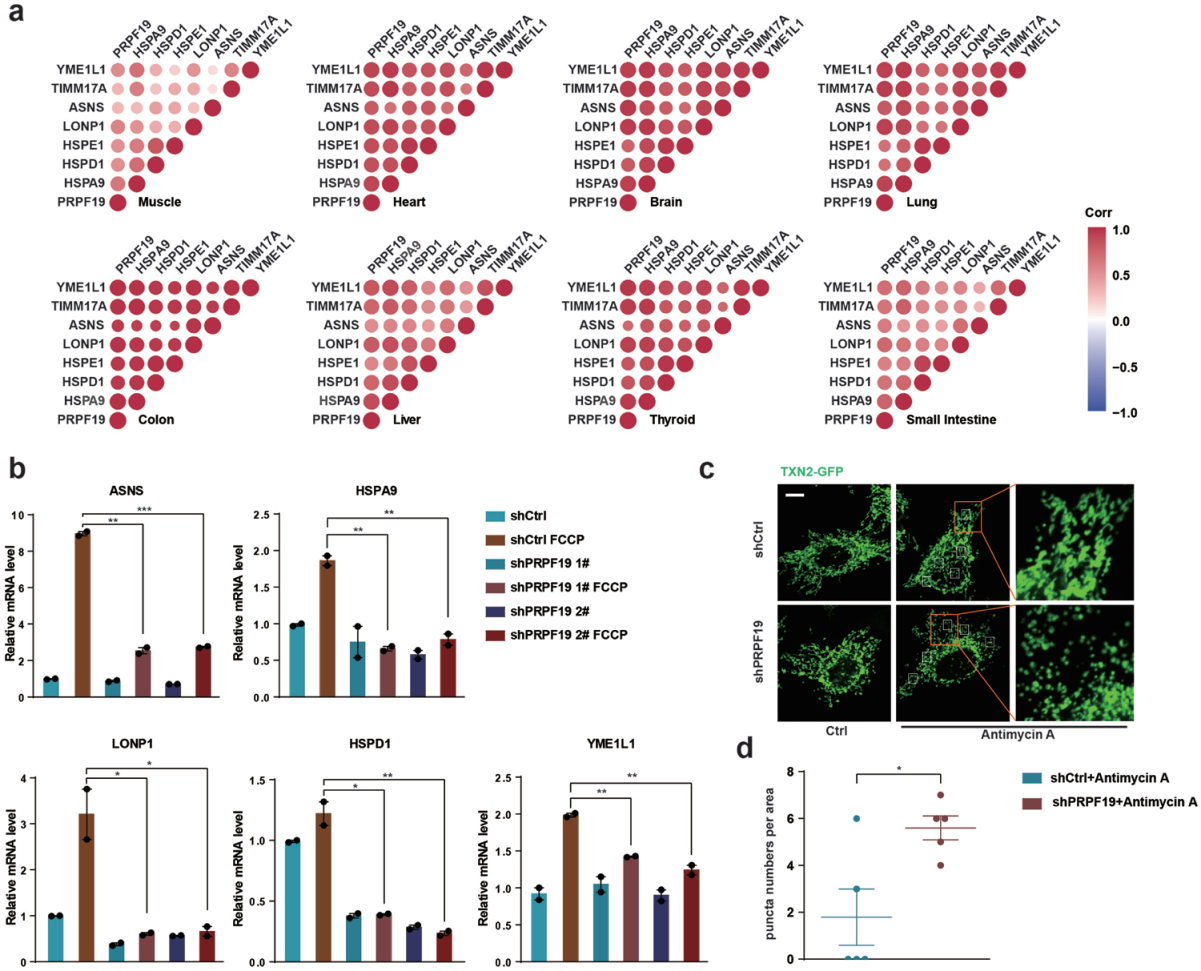
PRPF19 regulates UPR^mt^ in mammalian cells. (a) Pearson’s correlation coefficients of the mRNA levels of *PRPF19* and UPR^mt^ genes (*HSPA9*, *HSPD1*, *HSPE1*, *LONP1*, *ASNS*, *TIMM17A*, *YME1L1*) in various human tissues. Red color indicates positive correlation, and blue color indicates negative correlation. A darker color intensity corresponds to a higher correlation coefficient. (b) qPCR measurement of endogenous *ASAN*, *HSPA9*, *LONP1*, *HSPD1*, and *YME1L1* mRNA levels in wild-type or *PRPF19* knockdown HEK293T cells. Cells were treated with FCCP (final concentration of 20 μM) for 12 h. *P < .05, **P < .005, ***P < .0005. (c) Mitochondrial morphology of HeLa cells stably expressing TXN2-GFP. HeLa cells were treated with control shRNA or shPRPF19 in the presence or absence of Antimycin A (10 μg/ml, 3 h). Right panels show zoomed areas (red squares). Scale bar, 10 µm. (d) Quantifications of punctate mitochondria in the same area (*n* = 5 smaller squares) of Hela cells indicated in Fig. 6c. *P < .05.

We further examined if PRP-19 plays a conserved role in modulating mitochondrial homeostasis in higher eukaryotes. Interestingly, the expression levels of PRPF19 (mammalian ortholog of PRP-19) strongly correlate with the mitochondrial proteases YME1L1 and LONP1, the mitochondrial import inner membrane translocase TIMM17A, the asparagine synthetase ASNS, and the mitochondrial chaperones HSPE1, HSPD1, and HSPA9 in various human tissues (Fig. 6a). To validate the function of PRPF19 in mediating mitochondrial homeostasis in mammals, we used shRNA to knock down *PRPF19* in HEK293T cells and examined the expression levels of mitochondrial stress response genes. Deficiency of PRPF19 suppressed the induction of mitochondrial stress response genes in cells treated with FCCP(Carbonyl cyanide 4-(trifluoromethoxy)phenylhydrazone) , a potent uncoupler of mitochondrial oxidative phosphorylation (Fig. 6b). Knockdown of *PRPF19* impaired both basal and ATP-linked respiration in the presence or absence of mitochondrial perturbation (Supplementary Fig. S3). In the presence of Antimycin A, mitochondrial morphology was disrupted more extensively in PRPF19 knockdown cells than in wild-type cells (Fig. 6c and d). Taken together, these results revealed an evolutionarily conserved role of PRPF19 in mediating mitochondrial stress response.

## Discussion

Upon mitochondrial perturbation, UPR^mt^ is activated to initiate a mitochondrion-to-nucleus crosstalk to activate the transcription of mitochondrial stress response genes, as well as innate immune response genes. Here, we report that splicing factors such as PRP-19 are required for the activation of UPR^mt^ in *C. elegans*. In addition, we show that PRPF19 (mammalian ortholog of PRP-19) also mediates mitochondrial homeostasis in mammals.

*prp-19* is an essential gene. *prp-19* RNAi causes sterility and embryonic lethality. Therefore, in several experiments (Figs 1c and 4c and Supplementary Fig. S1), we used the temperature-sensitive sterile *glp-4* mutant strain to avoid collecting the embryos generated in the control RNAi group, which may affect the results. Knockdown of *prp-19* suppresses the induction of both UPR^mt^ and UPR^ER^, but not the induction of the heat shock response (Fig. 1a–c, h–j), which reveals the specificity of PRP-19 in regulating stress responses. Knockdown of *prp-19* promotes the accumulation of polyQ35::YFP aggregates in muscles. We speculate that the effect of PRP-19 on age-associated polyQ accumulation is due to UPR^mt^ but not UPR^ER^ based on the following two reasons: (i) it has been shown that the accumulation of polyQ35::YFP aggregates in muscles is unaffected by the expression of *xbp-1*s, which activates UPR^ER^ [31], and (ii) our previous studies showed that knockdown of the histone deacetylase HDA-1, which specifically regulates UPR^mt^ but not UPR^ER^, promotes polyQ35::YFP aggregate accumulation in muscles [11].

It has been reported that PRP-19 has both pre-mRNA processing factor and E3 ubiquitin ligase activities [32]. The E3 ligase activity of PRP-19 is critical for the catalytic activation of the spliceosome [23]. On the other side, *Prp19* has been reported to require components of the Prp19C/NTC complex for the E3 activity [33]. Taking these findings together, it is very difficult to separate the putative E3 activity of PRP-19 from the splicing activity. We knocked down several splicing factors that function outside the Prp19C/NTC complex (Fig. 2b–d), and found that many of those factors also affect UPR^mt^. These results suggest that PRP-19 may modulate UPR^mt^ through its function in splicing.

Splicing has been shown to occur and function co-transcriptionally. Although PRP-19 and other splicing factors modulate the activation of UPR^mt^, we did not observe extensive AS events when *C. elegans* was challenged with mitochondrial perturbation (Supplementary Fig. S1a). Therefore, the splicing machinery might regulate transcription through a splicing-independent mechanism. At this stage, how the splicing machinery specifically modulates the transcription of mitochondrial stress response genes upon mitochondrial inhibition remains speculative. We speculate that PRP-19 functions downstream of ATFS-1 based on the following reasons: (i) knockdown of *prp-19* impairs the induction of the UPR^mt^ gene *hsp-60* driven by the expression of ATFS-1^Δ1-32^, (ii) knockdown of *prp-19* impairs the induction of ATFS-1-dependent UPR^mt^ genes, (iii) lack of PRP-19 does not affect the binding of ATFS-1 to the promoters of UPR^mt^ genes, and (iv) knockdown of other splicing factors besides PRP-19 also impairs the induction of UPR^mt^ genes. Notably, ATFS-1-dependent transcription of *hsp-6* is basally active throughout worm development, and it can be further activated by diverse mitochondrial perturbations. If PRP-19 acts downstream of ATFS-1 to modulate the transcription of UPR^mt^ genes, knockdown of *prp-19* by RNAi may suppress the basal expression level of *hsp-6* under normal conditions (Fig. 1c). Currently, we do not fully understand how PRP-19 and other splicing factors modulate ATFS-1-dependent transcription. One possibility is that splicing factors may specifically interact with both ATFS-1 and the C-terminal domain of RNA Polymerase II under mitochondrial stress conditions to promote the transcriptional elongation of UPR^mt^ genes [21, 22]. Further analysis comparing ChIP-seq results using antibodies against RNA Polymerase II and PRP-19 may provide evidence of how splicing factors regulate the transcription of UPR^mt^ genes. Nucleosome positioning experiments may also shed light on the effect of PRP-19 on transcription. However, it is also possible that PRP-19 may function in parallel to ATFS-1.

## Materials and methods

### Materials

Reagent and resource information is listed in Table 1.

**Table 1:** Information for key reagents and resources.

Reagent or resource	Source	Identifier
Antibodies		
Rabbit monoclonal anti-HA antibody	Cell Signaling Technology	Cat# 3724; RRID: AB_1549585
Rabbit polyclonal anti-GFP antibody	Abcam	Cat# ab290; RRID: AB_303395
Rat monoclonal anti-alpha Tubulin antibody	Abcam	Cat# ab64332; RRID: AB_1140548
Rabbit monoclonal anti-ACTB antibody	ABclonal	Cat# AC026 RRID: AB_2768234
Bacterial and virus strains		
Trans1-T1	Transgen	Cat# CD501-03
TransStbl3	Transgen	Cat# CD521-02
*Pseudomonas aeruginosa*	Pellegrino *et al*. [7]	PA14
Chemicals, peptides, and recombinant proteins		
Sodium azide	Sigma	Cat# S8032
Isopropyl-β-d-thiogalactoside	Amresco	Cat# 0487-100G
Antimycin A	Sigma	Cat# A8674
Carbonyl cyanide 4 (trifluoromethoxy)phenylhydrazone	MedChemExpress	Cat# HY-100410
NuPAGE LDS loading buffer	Thermo Fisher	Cat# NP0007
Pierce™ Anti-HA Magnetic Beads	Thermo Fisher	Cat# 88837
GFP-Trap Agarose Beads	ChromoTek	Cat# gta-20
Critical commercial assays		
One-Step gDNA Removal and cDNA Synthesis SuperMix	Transgen	Cat# AE311-02
SYBR Green PCR Master Mix	Bio-Rad	Cat# 1725121
ChIP DNA Clean & Concentrator	Zymo	Cat# D5205
XF Cell Mito Stress Test Kit	Agilent	Cat# 103015100
Pierce™ Rapid Gold BCA	Thermo Fisher	Cat# A53226
Deposited data		
ATFS-1 CHIP-seq data	Nargund *et al*. [6]	GEO: GSE63803
Raw and analyzed data	This paper	GEO: GSE191294
Human tissue RNA-seq data	GTEx	https://www.gtexportal.org/home/datasets
Experimental models: cell lines		
Human: HEK293T cells	ATCC	Cat# CRL-3216
Human: Hela	ATCC	Cat# CRM-CCL-2
Experimental models: *C. elegans* strains		
zcls13[*hsp-6::gfp*]	CGC	SJ4100
zcls4[*hsp-4p::gfp*]	CGC	SJ4005
dvls70[*hsp-16.2::gfp*]	CGC	CL2070
zcls39[*dve-1p::dve-1::gfp*]	CGC	SJ4197
agls17[*irg-1p::gfp*]	CGC	AU133
rmls132[*unc-54p::Q35::yfp*]	CGC	AM140
*glp-4(bn2)*	CGC	SS104
*N2*	CGC	N2
*isp-1(qm150)*	CGC	MQ887
liuls1[*hda-1p::hda-1::flag::ha; odr-1p::dsRed*]	YSL1	N/A
liuls2[*hda-1p::hda-1::gfp; odr-1p::dsRed*]	YSL2	N/A
liuls1[*hda-1p::hda-1::flag::ha; odr-1p::dsRed*]; zcls39[*dve-1p::dve-1::gfp*]	YSL3	N/A
*glp-4(bn2);* rmls132[*unc-54p::Q35::yfp*]	YSL8	N/A
liuEx3[*hsp-16.2p:: atfs-1*^*Δ1-32*^*::gfp;odr-1p::dsRed*]	liuEx3	N/A
liuEx4[*prp-19p::mcherry::prp-19; mec-7p::yfp;* zcls13[*hsp-6p::gfp*]]	liuEx4	N/A
*atfs1(tm4525);hsp16p::atfs1*^*Δ132.myc*^*;hsp-60p::gfp; myo-3::mCherry*	Dr. Cole Haynes	N/A
*hsp-16.2p::atfs-1*^*Δ1-32.myc*^*::gfp*	Dr. Cole Haynes	N/A
Oligonucleotides		
*hsp-6* QPCR forward: 5ʹ–3ʹ TCCCAAGTCTTCTCTACCGC	This paper	N/A
*hsp-6* QPCR reverse: 5ʹ–3ʹ CACGATCTCTGGCTGAAACG	This paper	N/A
*hsp-60* QPCR forward: 5ʹ–3ʹ GAGAGAAGAAGGACCGTGTC	This paper	N/A
*hsp-60* QPCR reverse: 5ʹ–3ʹ GACTTCATCAATAATCGACGATGG	This paper	N/A
*act-3* QPCR forward: 5ʹ–3ʹ TCCCTCGAGAAGTCCTACGA	This paper	N/A
*act-3* QPCR reverse: 5ʹ–3ʹ TCCTGGGTACATGGTGGTTC	This paper	N/A
*prp-19* QPCR forward: 5–-3ʹ TTGGCTACGGGATCAGAGGA	This paper	N/A
*prp-19* QPCR reverse: 5ʹ–3ʹ CACGTCACCAATGAACGAGC	This paper	N/A
*mul-1* QPCR forward: 5ʹ–3ʹ ATACACCTGTGGAACCTGCG	This paper	N/A
*mul-1* QPCR reverse: 5ʹ–3ʹ ACGGTCTGGGAGGAGCTAAT	This paper	N/A
*clec-4* QPCR forward: 5ʹ–3ʹ GAGCGACACTGGTGACTGTG	This paper	N/A
*clec-4* QPCR reverse: 5ʹ–3ʹ CCATCCAGAATAGGTTGGCG	This paper	N/A
*clec-265* QPCR forward: 5ʹ–3ʹ CACCACACCCCTCACGTATG	This paper	N/A
*clec-265* QPCR reverse: 5ʹ–3ʹ GAGAATCTGGGCATGGCTGA	This paper	N/A
*K08D8.5* QPCR forward: 5ʹ–3ʹ TGTGCCGGAAATACGCTGAT	This paper	N/A
*K08D8.5* QPCR reverse: 5ʹ–3ʹ TAGCGTACGTTCCCTGAGGA	This paper	N/A
*pals-23* QPCR forward: 5ʹ–3ʹ AGGAAACGCTAAGCCAACCA	This paper	N/A
*pals-23* QPCR reverse: 5ʹ–3ʹ AGTTCTGGAGGAACACGAGC	This paper	N/A
*rpl-32*QPCR forward: 5ʹ–3ʹ CGTTTCCGGAACCAAGGTCA	This paper	N/A
*rpl-32*QPCR reverse: 5ʹ–3ʹ GTCTGCGGACACGGTTATCA	This paper	N/A
*hsp-4*QPCR forward: 5ʹ–3ʹ ATTGAGTGGCTCGGAAGCAA	This paper	N/A
*hsp-4* QPCR reverse: 5ʹ–3ʹ GAACCCGATGGCAGCAAGTA	This paper	N/A
*atp-2*QPCR forward: 5ʹ–3ʹ TCTCGAGGTAGTCGGACGTT	This paper	N/A
*atp-2*QPCR reverse: 5ʹ–3ʹ GAGTTTCTGGTCCGACTGGG	This paper	N/A
*prp-19*(codon-adapted) QPCR forward: 5ʹ–3ʹ CCCTCTGCAAGGTCTCTGTC	This paper	N/A
*prp-19*(codon-adapted) QPCR reverse: 5ʹ–3ʹ CTTGACCTCTCCGTCCTCAG	This paper	N/A
*hsp-6* ChIP-QPCR forward: 5ʹ–3ʹ GCATCATTATTCTCCTAAAACTTG	This paper	N/A
*hsp-6* ChIP-QPCR reverse: 5ʹ–3ʹ GCTTTTCAACCGTTAAACGGG	This paper	N/A
*hsp-60* ChIP-QPCR forward: 5ʹ–3ʹ TCTCGTGCGGCAAACTTG	This paper	N/A
*hsp-60* ChIP-QPCR reverse: 5ʹ–3ʹ ATGGCTAATTTACATCAGAATAGACT	This paper	N/A
*mrpl-2* ChIP-QPCR forward: 5ʹ–3ʹ GAGAATCCAGCTACCCAAATCGACG	This paper	N/A
*mrlp-2* ChIP-QPCR reverse: 5ʹ–3ʹ CACAGGGCATTTGTACCTTCCTCC	This paper	N/A
*C12D12.1* Exon6 forward: 5ʹ–3ʹ CACAGGGCATTTGTACCTTCCTCC	This paper	N/A
*C12D12.1* Exon6 reverse: 5ʹ–3ʹ CGGTACTTGGTGGTGATGTAGGC	This paper	N/A
*C12D12.1* SE forward: 5ʹ–3ʹ GTAAGCAAACCAATACCATATCACCACATC	This paper	N/A
*C12D12.1* SE reverse: 5ʹ–3ʹ CTGAATTAGTAGTTACTACAGGCTTAGTTGTG	This paper	N/A
*C53B7.5* SE forward: 5ʹ–3ʹ CTGGATCCACTTGCCGTTACTC	This paper	N/A
*C53B7.5* SE reverse: 5ʹ–3ʹ TTAAGAGCTTCTCTTTTGTCTGAGCACG	This paper	N/A
ACTB QPCR forward: 5ʹ–3ʹ GTCATCACCATTGGCAATGAG	This paper	N/A
ACTB QPCR reverse: 5ʹ–3ʹ CGTCATACTCCTGCTTGCTG	This paper	N/A
HSPD1 QPCR forward: 5ʹ–3ʹ CAGTCAAGGCTCCAGGGTTT	This paper	N/A
HSPD1 QPCR reverse: 5ʹ–3ʹ TGGCATCGTCTTTGGTCACA	This paper	N/A
ASNS QPCR forward: 5ʹ–3ʹ ATCACTGTCGGGATGTACCC	This paper	N/A
ASNS QPCR reverse: 5ʹ–3ʹ TGATAAAAGGCAGCCAATCC	This paper	N/A
ShPRPF19 1# forward: 5ʹ–3ʹ CCGGCCTCAAGTTCTACAGCCTGTACTCGAGTACAGGCTGTAGAACTTGAGGTTTTT	This paper	N/A
ShPRPF19 2# forward: 5ʹ–3ʹ CCGGCGGCTCATCGAGAAGTACATTCTCGAGAATGTACTTCTCGATGAGCCGTTTTT	This paper	N/A
LONP1 QPCR forward: 5ʹ–3ʹ CAAGCAGACCCACCGTAAGT	This paper	N/A
LONP1 QPCR reverse: 5ʹ–3ʹ CAGCTCCTCGTCCACAACAT	This paper	N/A
YME1L1 QPCR forward: 5ʹ–3ʹ CCCAGGGACTGGAAAGACAC	This paper	N/A
YME1L1 QPCR reverse: 5ʹ–3ʹ GAGCATTCGCCTTTGCTTCC	This paper	N/A
HSPA9 QPCR forward: 5ʹ–3ʹ TGGTGAGCGACTTGTTGGAAT	This paper	N/A
HSPA9 QPCR reverse: 5ʹ–3ʹ ATTGGAGGCACGGACAATTTT	This paper	N/A
Recombinant DNA		
pBOBI vector	Laboratory of Prof. Sheng-Cai Lin	Zhang *et al*. [34]
PLKO.1 vector	Addgene	Cat#8453
Software and algorithms		
GraphPad Prism	GraphPad Software	https://www.graphpad.com
rMATS 3.2.5	Xing Lab	http://rnaseq-mats.sourceforge.net/rmats3.2.5/
R 4.0.3	N/A	http://www.r-project.org/

*Resource availability*: Further information and requests for resources and reagents should be directed to and will be fulfilled by the lead contact, Ying Liu (ying.liu@pku.edu.cn).

N/A, Not Applicable.

### Experimental model and subject details

Worms were maintained on Nematode Growth Medium (NGM) plates seeded with OP50 bacteria under normal conditions and grown on RNAi plates supplemented with 1.2 mg/ml Isopropyl β-D-thiogalactoside (IPTG) in NGM plates seeded with RNAi bacteria. HEK293T cells and HeLa cells were obtained from ATCC. Cells were cultured in DMEM high glucose medium supplemented with 10% (v/v) fetal bovine serum at 37°C.

### Methods

#### RNA interference

All the RNAi bacteria were obtained from the Ahringer library. RNAi bacteria were grown in liquid lysogeny broth (LB) medium containing 50 μg/ml carbenicillin at 37°C overnight. IPTG (0.2 μg/ml) was added to the RNAi bacterial culture and incubated at 37°C for 4 h to induce the expression of double-strand RNA (dsRNA). Concentrated RNAi bacteria (25×) were seeded onto RNAi plates with 1.2 mg/ml IPTG. Synchronized L1 worms were raised on the RNAi plates at 20 or 25°C.

For shRNA knockdown in mammalian cells, 5 × 10^5^ HEK293T cells in six-well plates were transfected with the plasmid mixture containing 1.5 μg pLKO.1, 0.9 μg psPAX, and 0.6 μg pMD2.G. Medium was changed 12 h after transfection. Transfected cells were cultured for another 36 h to allow lentivirus production. Cells (5 × 10^5^) in 12-well plates were cultured in a virus-containing medium supplemented with 8 μg/ml polybrene for 24 h. Then, 1–2.5 μg/ml puromycin was added to the culturing medium for positive selection.

#### Induction of UPR^mt^

To induce UPR^mt^ with Antimycin A, synchronized L1 worms were raised on 6-cm RNAi plates at 20°C for 48 h. Four hundred microliters of M9 buffer containing 10 μg Antimycin A were dropped to the surface of worm plates. Fluorescent images were taken after another 24 h.

To induce UPR^mt^ with RNAi bacteria, synchronized L1 worms were raised on RNAi plates seeded with indicated RNAi bacteria at 20°C (N2) or 25°C (*glp-4(bn2)*) for 24 h. Secondary RNAi bacteria (*cco-1*, *atp-2*, or *spg-7* RNAi) pre-induced with IPTG were then added to the plates. Worms were collected or imaged after 48 h.

To induce UPR^mt^ in mammalian cells, cells were treated with 20 μM FCCP for 12 h.

#### Induction of UPR^ER^ and heat shock response

To induce UPR^ER^ with *hsp-4* RNAi, synchronized L1 worms were grown on RNAi plates seeded with the indicated RNAi bacteria at 20°C for 24 h. *hsp-4* RNAi bacteria pre-induced with IPTG were then added to the plates. Fluorescence images were taken after another 48 h.

To induce UPR^ER^ with tunicamycin, synchronized L1 worms were grown on RNAi plates seeded with control RNAi bacteria, or *prp-19* RNAi bacteria diluted with control RNAi for 72 h. Gravid adult animals were bleached and the eggs were collected. Synchronized L1 worms were then grown on RNAi plates seeded with the indicated RNAi bacteria in the presence or absence of tunicamycin (10 μg/ml NGM) at 20°C. Worms were collected after 48 h.

To induce heat shock response, synchronized L1 worms were raised on RNAi plates at 20°C for 24 h. The worms were then raised at 37°C for 1 h and transferred back to 20°C. Worms were imaged after 24 h.

#### Microscopy

Worms were placed on 2% agarose pads in 100 mM NaN_3_ diluted with M9 buffer. The images were taken with a Zeiss Imager M2 microscope. HeLa cells stably expressing TXN2-GFP were treated with 10 μg/ml Antimycin A for 3 h to induce mitochondrial stress. Cells were washed with pre-cold phosphate-buffered saline (PBS) once and fixed with 4% paraformaldehyde fix solution for 10 min. Mitochondrial morphology images were taken with a Zeiss 880 Airyscan FAST microscope.

#### Western blotting

Worms cultured on 6-cm plates under the indicated conditions (~1000 worms) were collected and washed several times with M9 buffer until the supernatant was clear. Worms were centrifuged and worm pellets were suspended with 4× NuPAGE LDS loading buffer. The samples were then boiled at 95°C for 10 min. Proteins were separated on 10% sodium dodecyl sulfate–polyacrylamide gel electrophoresis (SDS–PAGE) gels and transferred onto polyvinylidene fluoride (PVDF) membranes. Membranes were then blocked with 5% milk in PBST (1% Tween-20 in PBS) followed by incubation with primary and secondary antibodies.

#### RNA isolation and quantitative RT-PCR

Worms raised on 6-cm plates under the indicated conditions (~1000 worms) were harvested and washed with M9 buffer for 20 min until the supernatant was clear. Worms were centrifuged and worm pellets were suspended with 1 ml TRIzol reagent. Repeated freeze-thawing of worm samples was performed with liquid nitrogen five to six times before RNA isolation. HEK293T cells in 12-well plates were collected with PBS and suspended with 1 ml TRIzol reagent. RNA was isolated using chloroform extraction, precipitated with isopropanol, and washed with 75% ethanol and 100% ethanol. cDNA was synthesized with One-Step gDNA Removal and cDNA Synthesis SuperMix. qPCR was carried out using SYBR Green PCR Master Mix. For quantification, transcript levels were normalized to *act-3* or *rpl-32* for worms and ACTB for mammals.

#### Immunoprecipitation

Approximately, 50 000 synchronized L1 worms were raised at 20°C on 15-cm RNAi plates seeded with pre-induced control RNAi or *prp-19* RNAi bacteria. Late L4 worms were collected with M9 buffer and washed several times until the supernatant was clear. Worms were centrifuged and worm pellets were suspended in 1 ml IP lysis buffer [50 mM Tris-HCl pH 7.5, 150 mM NaCl, 1% NP40, 1 mM ethylenediaminetetraacetic acid (EDTA), 1 mM ethylene glycol tetraacetic acid (EGTA), 20 mM *N*-ethylmaleimide, and proteinase inhibitor cocktail] and ground in a Dounce Tissue Grinder on ice for 80 times. Worm lysates were centrifuged at 20 000× g for 15 min at 4°C. The supernatants were saved and protein lysate quantification was performed using the Pierce bovine serum albumin (BSA) kit. Immunoprecipitation was performed by mixing protein lysate with 15 μl pre-washed Anti-HA magnetic beads and rotating at 4°C for 4 h. Beads were then washed three times with 1 ml IP lysis buffer. Beads were collected, suspended with 15 μl 4× NuPAGE LDS loading buffer, and boiled at 95°C for 15 min.

#### Lifespan analysis

More than 100 synchronized L1 worms were raised on RNAi plates seeded with control RNAi or *prp-19* RNAi and cultured at 20°C for 24 h. *atp-2* RNAi bacteria pre-induced with IPTG were added for another 48 h. Worms were transferred every 2 days to new RNAi plates seeded with corresponding RNAi bacteria. Lifespan experiments were performed in two independent biological experiments.

More than 100 synchronized L1 *isp-1* mutant worms were raised on RNAi plates seeded with control RNAi or *prp-19* RNAi and cultured at 20°C for 48 h. Worms were counted and transferred every day to new RNAi plates seeded with the corresponding RNAi bacteria. Lifespan experiments were performed in two independent biological experiments.

#### PA14 slow-killing assay

*Pseudomonas aeruginosa* (PA14) was cultured in 5 ml liquid LB containing 50 μg/ml ampicillin at 37°C overnight. Eighty microliters of the bacterial solution were dropped in the center of 3.5-cm slow-killing plates and incubated at 37°C for 24 h. The plates were then transferred to 25°C and incubated for another 24 h. Synchronized L1 *glp-4* (*bn2*) worms were grown at 25°C on RNAi plates seeded with control RNAi or *prp-19* RNAi. L4 worms were picked and transferred to the slow-killing plates at 25°C. The number of dead nematodes in the plates was counted every 12 h. Three biological replicates of the PA14 slow-killing assay were performed per condition.

#### Induction of nematode immune response

*Pseudomonas aeruginosa* (PA14) was cultured in 5 ml liquid LB containing 50 μg/ml ampicillin at 37°C overnight. Four hundred microliters of the bacterial solution were dropped into 6-cm slow-killing plates and incubated at 37°C for 24 h. The plates were then transferred to 25°C for another 24 h. L4 worms (N2) were washed off the plates after being treated with the indicated RNAi from L1, transferred to the slow-killing plates, and placed at 25°C for 2 h. Worms were harvested and washed with M9 buffer until the supernatant was clear. Worm pellets were then suspended in a 1 ml TRIzol reagent and prepared for RNA extraction.

#### Mobility assay

unc-54p::*Q35::yfp* animals were touched twice lightly with platinum wire on the tail. The total number of animals and those who could not move or change their positions after touching were counted on days 1, 7 and 11 of adulthood. Worms were randomly selected. Three biological replicates of the mobility assay were performed under each condition.

#### Chromatin immunoprecipitation

ChIP assay was performed using the *hsp-16.2p::atfs-1*^*Δ1-32.myc*^*::gfp* strain. Synchronized L1 worms were raised in 15-cm RNAi plates containing control RNAi or *prp-19* RNAi at 16°C until the worms reached the L4 stage. Animals were cultured at 37°C for 1.5 h and returned to 16°C for an additional 2 h. Approximately, 450 000 worms in each condition were collected with M9 buffer containing 0.01% Triton-X100. Worms were then washed with M9 buffer and pre-cooled PBS until the supernatant was clear.

After centrifugation at 2000 rpm for 1 min, worm pellets were suspended with 1 ml formaldehyde cross-linking solution (PBS containing 2% formaldehyde) and fixed at room temperature for 15 min. Glycine solution was added to the mixture to reach a final concentration of 125 mM and incubated at room temperature for 5 min. The mixture was centrifuged at 2000 rpm for 4 min and the pellets were washed with chilled PBS containing proteinase inhibitors three times. Two milliliters of SDS lysis buffer (1% SDS, 0.01 M EDTA pH 8.0, proteinase inhibitor cocktail) were added to the worm pellets and the mixture was placed on ice for 10 min. The animals were then fully ground with a homogenizer until no intact worms could be observed under a stereomicroscope. The worm lysates were fully sonicated (30% sonication power, cycled in a program of 10 s sonication and 10 s pause, 20 cycles). Lysates were centrifuged at 20 000 × *g* for 15 min at 4°C and the supernatants were collected. The protein lysates were quantified using the Pierce BSA kit. A small amount of each supernatant was taken as the input sample. Eight milliliters of ChIP dilution buffer (1% SDS, 11% Triton-X100, 1.2 mM EDTA pH 8.0, 16.7 mM Tris-HCl, 16.7 mM NaCl, proteinase inhibitor cocktail) were added to the supernatant. Half the volume of each worm lysate was incubated with GFP-Trap agarose (pre-coated with 5% BSA and 400 μg/ml *Siniperca chuatsi* sperm DNA) and the other half volume was incubated with control agarose beads. The mixture was rotated at 4°C for 4 h.

After incubation, beads were washed once with low salt washing buffer (1% SDS, 1% Triton-X100, 2 mM EDTA pH 8.0, 20 mM Tris-HCl pH 8.0, 150 mM NaCl), once with high salt washing buffer (1% SDS, 1% Triton-X100, 2 mM EDTA pH 8.0, 20 mM Tris-HCl pH 8.0, 500 mM NaCl), once with LiCl washing buffer (0.25 mM LiCl, 1% IGEPAL-CA630, 1% sodium deoxycholate, 1 mM EDTA, 10 mM Tris pH 8.1) and finally twice with TE solution. After centrifugation at 1000 × *g* for 1 min, the beads were mixed with 230 μl freshly prepared elution buffer (1% SDS and 0.1 M sodium bicarbonate) and incubated at room temperature for 20 min. The mixture was centrifuged at 20 000 × *g* for 15 min and the supernatant was saved. Eight microliters of 5 M NaCl were added to the supernatant and de-cross-linked at 60°C for 8 h. For input samples, 1 μl of 5 M NaCl solution was added for every 20 μl sample. Four microliters of 0.5 M EDTA, 8 μl of 1 M Tris-HCl, pH 6.5, and 1 μl of proteinase K were added to the immunoprecipitation samples after de-cross-linking. Two microliters of 0.5 M EDTA, 4 μl of 1 M Tris-HCl pH 6.5, and 1 μl of proteinase K were added to the input samples, and digested at 45°C for 2 h. The DNA fragments were recovered using a ChIP DNA Clean & Concentrator kit.

#### Mitochondrial respiration analysis

Mitochondrial respiration was measured using an Agilent Seahorse XFe24 analyzer and Seahorse XF Cell Mito Stress Test Kit (Agilent). Briefly, 4 × 10^4^ control or PRPF19 knockdown HEK293T cells were placed per well with three replicates in Seahorse XF24 cell culture microplates (Agilent). Cells were grown overnight and treated with 10 nM Antimycin A for 30 min. The subsequent assay was performed according to the Agilent Seahorse manufacturer’s instructions. The final reports of OCR signals were generated using GraphPad software.

#### RNA-seq sample preparation and RNA-seq data analysis

Synchronized L1*glp-4* (*bn2*) worms were raised on RNAi plates seeded with control RNAi or *prp-19* RNAi bacteria at 25°C for 24 h. *atp-2* RNAi bacteria pre-induced with IPTG were then added. Worms were collected after an additional 48 h.

Differential AS events were analyzed with the rMATS tool (version: 3.2.5. Parameters: -t paired -len 101 -a 8 -c 0.05 -analysis U) based on the RNA-seq data from the indicated conditions. The sequencing reads were mapped using STAR (version: 2.7.5c). Genome version cell and gene annotations from Ensembl Release 87 (WBcel235.87) were used. The AS events were classified into five types: SE, RI, MXEs, A5SS, and A3SS. The number of significant AS events was detected using Junction Counts only mode by threshold false discovery rate (FDR) <0.05 and percent spliced-in (|ΔPSI|) >0.05.

For differential expression analysis, raw sequencing data were aligned to the ce11 reference genome using HISAT2 (version 2.1.0). Gene expression levels were quantified by feature Count (version 1.6.3) with the genomic annotation from UCSC (http://genome.ucsc.edu/cgi-bin/hgTables). Downstream analysis was performed using R (version 4.0.5). Differential gene expression analysis was performed by DESeq2. Genes with log_2_(fold change) >1 and adjusted *P*-value <0.05 were considered as differentially expressed genes. For clustering and heat map plots, we used the Pheatmap R package. Worm ATFS-1 ChIP-seq processed data [6] were obtained under accession GEO: GSE63803. The ATFS-1 binding genes were defined as the nearest genes to ATFS-1 ChIP-seq peaks.

#### Expression correlation analyses

Expression correlation analysis was performed using the expression data of PRPF19 and UPR^mt^-related genes in various human tissue samples from the GTEX database (https://www.gtexportal.org/home/datasets). Gene expression levels in the above samples were compared by calculating Pearson’s correlation. Heat map presentation was performed by the ggplot2 function package in R language.

#### Quantification and statistical analysis

The experiments in this paper were all repeated at least three times unless otherwise indicated. Statistical analysis was performed using GraphPad software and Student’s *t-*test (two-tailed, unpaired) to calculate the *P* values. IGV software was used to visualize the RNA-seq sequencing results.

## Supplementary Material

loac009_suppl_Supplementary_Material

loac009_suppl_Supplementary_Table_S1

## Data Availability

The accession number for the sequencing data reported in this paper is GEO:GSE191294.
