# Supplementary material for: Splicing factor PRP-19 regulates mitochondrial stress response
Source: Life Metab. 2022 Jun 24;1(1):81–93. doi: 10.1093/lifemeta/loac009 (PMC11749837; doi:10.1093/lifemeta/loac009)
Supplement: loac009_suppl_Supplementary_Material [file loac009_suppl_Supplementary_Material.pdf]

Supplementary Information

Splicing factor PRP-19 regulates mitochondrial stress response

Peixue Xia[1,2], Liankui Zhou[1,2], Jialiang Guan[4], Wanqiu Ding[1], and Ying Liu[1,2,3]*

a

AS events	Control + *atp-2* VS Control	*prp-19* VS Control	*prp-19* + *atp-2* VS Control + *atp-2*
A3SS	1(12.5%)	34(29.1%)	25(24.5%)
A5SS	3(37.5%)	13(11.1%)	7(6.9%)
MXE	0	7(6%)	3(2.9%)
RI	1(12.5%)	15(12.8%)	15(14.7%)
SE	3(37.5%)	48(41%)	52(51%)
Total	8	117	102

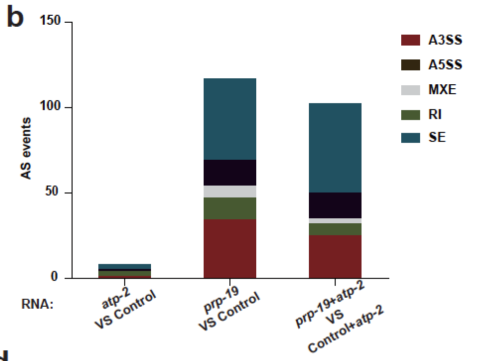

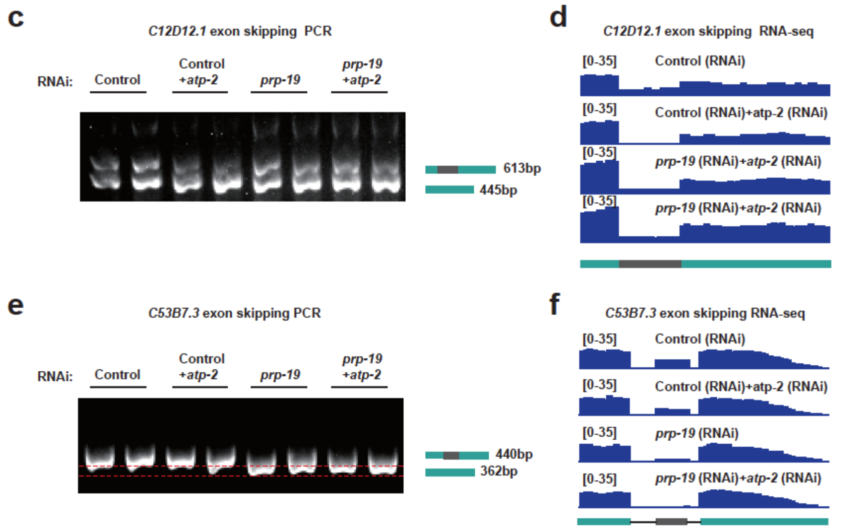

Supplementary Fig. S1 Suppression of *prp-19* affects the alternative splicing in *C. elegans*. a Knockdown of *prp-19* resulted in multiple alternative splicing events. Lists of differential alternative splicing events in worms raised on the indicated RNAi. FDR < 0.05 and |ΔPSI| > 0.05. **b** Histogram presentation of the results shown in (a). **c** Alternative splicing (exon 6 skipping) of endogenous *C12D12.1* in worms raised on the indicated RNAi was assessed by RT–PCR (two biological replicates). **d** Sequencing reads coverage for *C12D12.1*. **e** Alternative splicing (exon 5 skipping) of endogenous *C53B7.3* in worms raised on the indicated RNAi was assessed by RT–PCR (two biological replicates). **f** Sequencing reads coverage for *C53B7.3*.

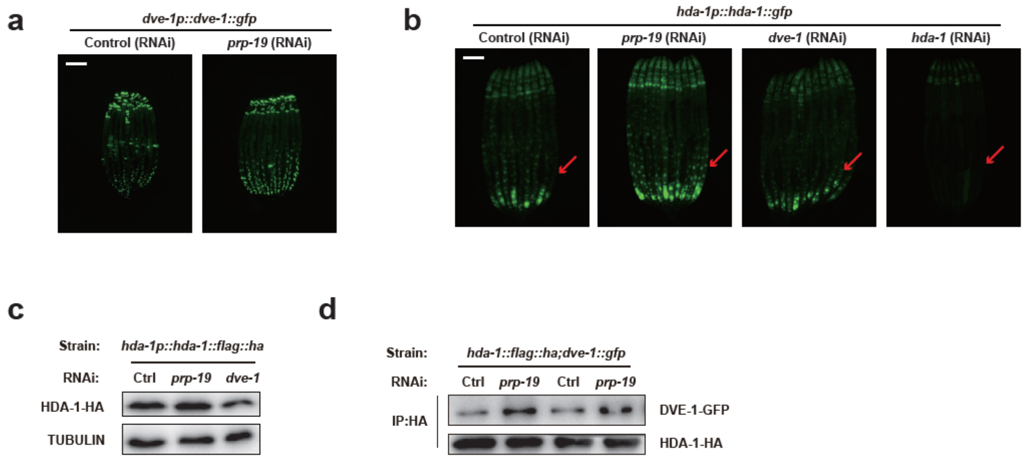

Supplementary Fig. S2 PRP-19 regulates UPR^{mt} independent of HDA-1 or DVE-1. a Fluorescence images of *dve-1p::dve-1::gfp* worms grown on control or *prp-19* RNAi. Scale bar, 200 µm. **b** Fluorescence images of *hda-1p::hda-1::gfp* worms. Worms were grown on control, *prp-19*, *dve-1* or *hda-1* RNAi. Scale bar, 100 µm. **c** Immunoblotting of HDA-1::FLAG::HA protein level in *hda-1p::hda-1::flag::ha* worms. Worms were raised on control, *prp-19* or *dve-1* RNAi. **d** Anti-HA immunoprecipitation of *hda-1p::hda-1::flag::ha*; *dve-1p::dve-1::gfp* worms. Worms were raised on control or *prp-19* RNAi.

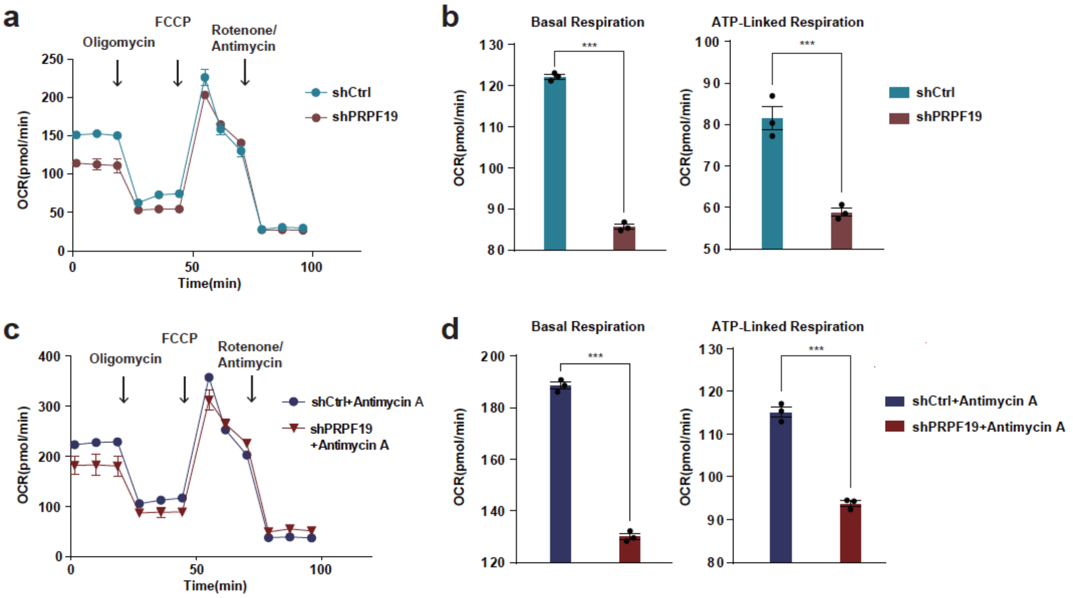

Supplementary Fig. S3 Knockdown of *PRPF19* impairs mitochondrial activity. a Mitochondrial respiratory status was measured in cells treated with control shRNA or shPRPF19. The final concentrations of Oligomycin, FCCP, Antimycin A and Rotenone are 1 μM, 2 μM, 1 μM and 1 μM. N=2 biological replicates for each condition. **b** Basal (left) and ATP-linked (right) mitochondrial respiration level in wild-type or PRPF19 knockdown HEK293T cells. ***p<0.0005. **c** Mitochondrial respiratory status of cells treated with control shRNA or shPRPF19 after Antimycin A treatment. Wild-type or PRPF19 knockdown cells were treated with 10 nM Antimycin A for 30 min. The final concentrations of Oligomycin, FCCP, Antimycin A and Rotenone are 1 μM, 2 μM, 1 μM and 1 μM. N=2 biological replicates for each condition. **d** Basal (left) and ATP-linked (right) mitochondrial respiration level in wild-type or PRPF19 knockdown HEK293T cells treated with Antimycin A. ***p<0.0005.

Supplementary Table S1 Differential alternative splicing events.
